# Mosquito cell lines: history, isolation, availability and application to assess the threat of arboviral transmission in the United Kingdom

**DOI:** 10.1186/1756-3305-7-382

**Published:** 2014-08-20

**Authors:** Thomas Walker, Claire L Jeffries, Karen L Mansfield, Nicholas Johnson

**Affiliations:** Animal Health and Veterinary Laboratories Agency, Woodham Lane, New Haw, Addlestone, Surrey, KT15 3NB UK; London School of Hygiene and Tropical Medicine, Keppel Street, London, WC1E 7HT UK

**Keywords:** Mosquitoes, Cell lines, Arboviruses, Vector competence

## Abstract

Mosquitoes are highly effective vectors for transmission of human and animal pathogens. Understanding the relationship between pathogen and vector is vital in developing strategies to predict and prevent transmission. Cell lines derived from appropriate mosquito hosts provide a relatively simple tool for investigating the interaction between the host and viruses transmitted by mosquitoes. This review provides a brief overview of the development of mosquito cell lines, methods of isolation, their availability and application for investigating insect-virus interactions.

## Background

Mosquitoes are responsible for the transmission of numerous infectious diseases including malaria, dengue fever, West Nile fever and Japanese encephalitis. Malaria is transmitted by *Anopheles* mosquitoes and results in the greatest mortality predominantly in Sub-Saharan Africa. In 2010, the World Health Organization estimated that there were 219 million cases (660,000 deaths) of malaria with 3.3 billion people at risk. Dengue is transmitted by *Aedes* mosquitoes and the incidence has grown dramatically with 50–100 million cases/year and 2.5 billion people at risk. Significant outbreaks of other mosquito-borne diseases such as Chikungunya, Japanese encephalitis and lymphatic filariasis impose a substantial burden on global health and economics in developing countries. Arboviral diseases transmitted by mosquitoes are a driver for poverty in much of the developing world and have shown an increase in incidence in the past few decades with major outbreaks occurring in previously non-endemic areas [1]. The geographical range of mosquito vector species, through changing environmental factors and international trade, has contributed to the increase in arboviral epidemics [2, 3]. Cost-effective treatment and prevention of mosquito-borne diseases is complicated by the diversity of pathogens, mosquito vector species and disease pathology. Strategies for prevention include vaccination, prophylaxis and vector control, although for some diseases such as dengue fever and West Nile fever, vector control is currently the only available strategy to prevent transmission.

**Figure 1:**
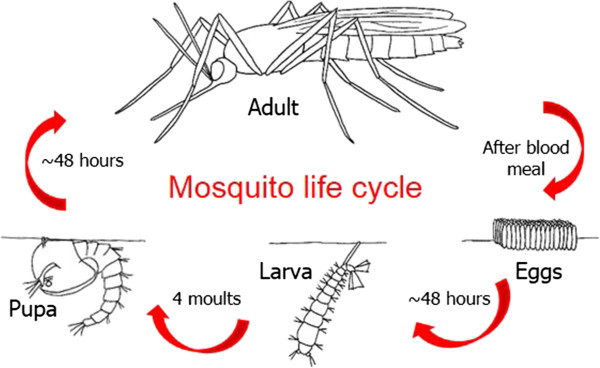
**An illustration of the lifecycle of mosquitoes and potential sources of cell lines.**

The mosquito lifecycle in its simplest form is composed of a series of life stages beginning with eggs laid on or near water that hatch after a number of days into larvae. The larvae obtain nutrition predominantly through filter-feeding but predation on other larvae and small invertebrates also occurs for certain species. Mosquitoes develop through four instar phases, to form non-feeding pupae, which metamorphose into adults (Figure 1). Adult males emerge first, followed by females and mating occurs when females are 2-3 days old. Whilst both males and females can derive nutrition from nectar, in most species females require a blood meal to promote egg development through the acquisition of protein and iron from blood. This provides the opportunity for pathogen transmission, particularly as female mosquitoes take multiple feeds during their lifecycle. Cells can be obtained from each of these developmental stages to generate cell lines appropriate for each experimental approach.

In recent years, interest has developed in a number of areas associated with virus-mosquito interactions. One key area of research has been elucidation of the immune response of insects against pathogens, and viruses in particular. In contrast to vertebrates, which have developed the interferon system to suppress virus replication, insects have an alternative innate immune mechanism commonly referred to as RNA interference (RNAi). This immune mechanism in mosquitoes has been reviewed extensively in recent years [4, 5]. Briefly, this virus control mechanism is stimulated by the presence of double-stranded RNA (an intermediary of virus replication) and leads to the recognition of specific sequences of single-stranded RNA (i.e. virus genomes) that are degraded by a cytoplasmic protein complex. Another area of research on mosquito-borne pathogens is to determine whether a given mosquito species is a potential vector for disease transmission. Vector competence studies can be undertaken in which infectious blood containing a pathogen is provided for the mosquito’s blood meal, followed by an assessment of pathogen development within the mosquito. Transmission of mosquito-borne diseases requires the pathogen to undergo a significant period of time within the mosquito vector called the extrinsic incubation period (EIP). When a female mosquito bites an infectious human or animal, the pathogen is ingested with the blood meal and disseminates from the mosquito midgut, eventually reaching the mosquito’s salivary glands for transmission to a new host. The time between ingestion of a blood meal and the ability to transmit virus is the EIP. The vectorial capacity of mosquitoes to transmit infectious pathogens depends on several factors including the EIP. For arboviruses such as dengue virus (DENV), the EIP is typically 7-14 days and external factors such as temperature can influence the EIP [6]. This experimental approach requires a range of skills and facilities including the ability to raise large numbers of mosquitoes (either colonised or wild caught), containment facilities for handling both the insect vector and the pathogen and expertise in both subjects. Therefore, alternative models to investigate vector-pathogen interactions are needed. *In vitro* studies using mosquito cell lines can be carried out to provide valuable information on aspects of these interactions. Here we review the history, isolation, availability and application of mosquito cell lines, and how cell lines can be used to contribute to understanding of the potential vector competence of UK mosquitoes for arboviruses.

## Review

### History of mosquito cell culture

There are over 500 insect cell lines now established from numerous insect orders including Diptera, Lepidoptera and Hemiptera, derived from different tissue sources [7, 8]. The major mosquito disease vector species are within the *Anopheles (An.)*, *Culex* (*Cx*.) and *Aedes (Stegomyia)* (*Ae*.) genera and several cell lines have been established. Table 1 contains a list of mosquito cell lines, which have been generated to our knowledge, with the associated disease transmitted by that species of mosquito. Early studies in the mid 1960s developed a generation of mosquito cell lines derived from larvae of the principal vector of DENV, *Ae. aegypti* [9]. The *Ae. aegypti* CCL-125 cell line was initially characterized as not susceptible to DENV [10]. However, more recent studies have come to the opposite conclusion and suggest that CCL-125 is permissive to DENV infection [11]. The Aag-2 cell line was also derived from *Ae. aegypti* in the late 1980s [12] and this cell line has been frequently used to study the mosquito immune system including the response to infection with Sindbis virus [13]. Subsequently *Ae. aegypti* cell lines such as RML-12 have been shown to be particularly susceptible to infection with DENV [14] and numerous other arboviruses including West Nile virus (WNV) and Japanese encephalitis virus (JEV) [15].

**Table 1:** **List of established mosquito cell cultures and associated mosquito-borne diseases**

***Mosquito species***	***Disease transmission***	***Cell line***	***Source***	***Reference***
*Aedes aegypti*	Dengue virus	CCL-125	Larvae	[10]
	Yellow fever virus	Aag-2	Embryos	[12]
		RML-12	Larvae	[14]
*Aedes albopictus*	Dengue virus	C6/36	Larvae	[10]
		C7-10	Larvae	[20]
*Aedes pseudoscutellaris*	Lymphatic filariasis	AP-61	Larvae	[14]
*Aedes triseriatus*	La Crosse encephalitis virus	A.t. GRIP-1	Embryos	[23]
		A.t. GRIP-2 & -3	Larvae	
*Aedes vexans*	West Nile virus	UM-AVE1	Embryos	[26]
	Rift Valley fever virus			
*Anopheles gambiae*	Malaria	Mos.55	Larvae	[27]
		Sua1B	Larvae	[28]
		4a-3B	Larvae	
*Anopheles stephensi*	Malaria	Mos.43	Larvae	[29]
		MSQ43	Larvae	[30]
*Anopheles albimanus*	Malaria	LSB-AA695BB	Embryos	[31]
*Culex quinquefasciatus*	West Nile virus	Unnamed	Ovaries	[32]
	Lymphatic filariasis	Unnamed	Embryos	[33]
*Culex theileri*	Rift Valley fever virus	Unnamed	Embryos	[34]
	Dog heartworm			
*Culex tritaeniorhynchus*	Japanese encephalitis virus	NIID-CTR	Embryos	[36]
*Culex bitaeniorhynchus*	Japanese encephalitis virus	Unnamed	Embryos	[37]
*Toxorhynchites amboinensis*	-	TRA-171	Larvae	[38]

The Asian tiger mosquito, *Ae. albopictus*, is a competent vector of many arboviruses including DENV, Chikungunya virus [16] and Eastern equine encephalitis virus (EEEV) [17]. The first cell lines developed from *Ae. albopictus,* such as the C6/36 cell line (originally known as the ATC-15 cell line), were generated from larvae in the mid 1960s [10]. Lineages of the C6/36 cell line have been widely used to study the relationship between arboviruses and mosquito vectors. Clones derived from the original cell line exhibited variable sensitivity to virus infection [18]. C6/36 cells were shown to be susceptible to a wide range of arboviruses, partially due to the lack of a functional RNAi response [19] and this cell line is now widely used to isolate arthropod-borne viruses. The C7-10 cell line was established from *Ae. albopictus* and has been shown to synthesize hormone-inducible proteins and thus forms a useful model for understanding hormone responses in insects [20].

*Ae. pseudoscutellaris* is a vector of subperiodic *Wuchereria bancrofti,* which is responsible for cases of human lymphatic filariasis in the South Pacific. A number of cell lines have been derived from larvae of this species in the 1970s, and were shown to be susceptible to a range of arboviruses [21]. In particular, an *Ae. pseudoscutellaris* cell line, AP-61, was established and shown to be susceptible to DENV infection [14, 22]. Another *Aedes* species, *Ae. triseriatus,* is a vector of La Crosse virus (LACV), which is a cause of encephalitis in the Midwest of the USA, and has been shown to be an efficient laboratory vector of EEEV. Three cell lines (A.t. GRIP-1, -2, and -3) were established from *Ae. triseriatus* embryos or larvae in the mid 1990s [23], and these cell lines have been shown to be susceptible to both LACV and snowshoe hare virus, a virus of wildlife that is exclusive to North America.

*Ae. vexans* mosquitoes are widespread throughout Europe and are potential vectors of WNV and RVFV [24, 25]. A cell line, UM-AVE1, has been established from embryos of *Ae. vexans* [26] but susceptibility to arboviral infection has not been reported.

*Anopheles* mosquitoes, particularly species in the *An. gambiae* complex, are responsible for malaria in Sub-Saharan Africa. Malaria has the highest mortality rate of any mosquito-borne disease and is justifiably the main focus of research on mosquitoes as vectors of disease. *Anopheles* cell lines have been generated including *An. gambiae* Mos.55 [27], Sua1B and 4a-3B from larvae [28]. The 4a-3B cell line is the first continuous insect cell line that produces prophenoloxidase, and is utilized as an *in vitro* model for the study of both the humoral and cellular immune defence of *An. gambiae* mosquitoes. *An. stephensi* mosquitoes are the primary vectors of malaria in South Asia, and several cell lines have been generated from larvae including Mos.43 [29] and MSQ43 [30]. *An. albimanus* is a major malaria vector in Central and South America, and the LSB-AA695BB cell line has been established from *An. albimanus* embryos [31].

There are a number of *Culex* mosquitoes that are responsible for transmission of human diseases. *Cx. quinquefasciatus* is a member of the *Cx. pipiens* complex and is the primary vector of Bancroftian lymphatic filariasis in tropical and sub-tropical regions. It is also the main vector of WNV in the USA and Europe. A cell line was established from this species using ovaries as the source tissue in the 1960s [32], and more recently a *Cx. quinquefasciatus* cell line has been established from embryos [33]. *Cx. theileri* is a natural vector of *Dirofilaria immitis* (dog heartworm) in southern Europe and is thought to be the major vector of Rift Valley fever virus (RVFV) in parts of southern Africa. A cell line was established for this vector species using embryos as the source material and was shown to be susceptible to a number of arboviruses [34]. *Cx. tritaeniorhynchus* mosquitoes are the principal vectors of JEV in Asia, and cell lines have also been established from embryos of this species [35, 36]. The *Cx. tritaeniorhynchus* NIID-CTR cell line is highly susceptible to both JEV and DENV infection, and provides a valuable model for virus replication in the host [36]. *Cx. bitaeniorhynchus,* a mosquito found throughout Asia and a potential vector of JEV, has been used to generate cell lines that are susceptible to several arboviruses [37].

*Toxorhynchites amboinensis* has no known vector competence for human pathogens as this mosquito is unable to blood feed. This species has been used in mosquito biocontrol strategies, since the large predatory larvae eat other mosquito larvae such as *Ae. aegypti*. However, a cell line derived from *Toxorhynchites amboinensis*, TRA-171, has been established and shown to be susceptible to DENV infection, although the significance of this observation remains unclear [38].

### Isolation and generation of primary cell line cultures

The source material for primary insect cell line culture is an important consideration. Ovaries were the first insect tissues used throughout the 1960s and 1970s, predominantly with Lepidoptera. Adult female mosquito tissues such as salivary glands or midguts could be used to generate cell lines that would be relevant to specific stages of virus-mosquito interactions that influence *in vivo* vector competence. For example, cell lines could be used to provide some preliminary information on the susceptibility of midgut or salivary gland cells prior to *in vivo* transmission studies. Embryos are now commonly used as the source for mosquito cell cultures as they contain cells with the potential to differentiate into larval and adult tissues, resulting in a wide diversity of cell morphologies.

A brief protocol for derivation of cells from mosquito embryos or tissues is detailed below.

- ■ The protocol for cell culture generation from either embryos or female tissues such as ovaries requires adult female mosquitoes (4-5 days old) to be blood fed using standard procedures. Mosquito eggs are collected 4 days post-blood feed, when an egg laying pot is placed into the cage containing gravid females.
- ■ Mosquito eggs are transferred from egg laying pots and rinsed thoroughly in 70% ethanol and then sterile water to prevent any bacterial contamination from the egg chorion (mosquito eggs are sufficiently impervious to simple disinfectants such as 70% ethanol). If tissues of adult female mosquitoes are used as the source material, tissues are dissected using sterile procedures.
- ■ Embryos or tissues are mechanically disrupted in insect cell culture media using a tissue homogenizer such as a plastic pestle. Additional methods have been used to disrupt embryonic cells such as the use of bleach or using enzymes including trypsin. However, care must be taken to avoid using chemicals that could potentially inhibit cell growth.
- ■ Insect cell culture media can vary for different mosquito species but standard formulations include Grace’s, Schneider’s and Mitsuhashi and Maramorosch media. A typical cell medium for mosquito cells is supplemented with fetal bovine serum and the antibiotics penicillin/streptomycin to prevent bacterial contamination.
- ■ The cell medium containing crushed mosquito material is then transferred to tissue culture flasks. Additional cell culture medium is added and cells are incubated at 26°C until cell attachment occurs. Culture medium is replaced every 3-4 days by vigorously shaking the tissue culture flasks and replacement with fresh medium.
- ■ Subcultures are obtained by passaging cells continually through disrupting confluent cell monolayers and transferring cells in suspension to a new tissue culture flask with fresh medium. Observation of cell morphology, division and growth with an inverted microscope is used to generate several subcultures.

A large number of embryos can be obtained from laboratory mosquito colonies to provide source material for multiple cell line culture experiments. An important consideration in the use of embryos for cell culture is the timing of embryogenesis. An incubation period after egg laying of 16-20 hours (approximately 66% of the time prior to larval hatching) was shown to be optimal for the successful generation of mosquito cell lines [31, 33]. A change in temperature during incubation has also been shown to facilitate establishment of primary cell cultures, possibly through stimulation of cell division [34].

### Availability of mosquito cell lines

The majority of cell lines currently used in arboviral research are obtained through direct contact with laboratories currently working with that particular cell culture. Only a few mosquito cell lines are commercially available. For example, the American Type Cell Collection only lists three cell lines derived from *Ae. aegypti*, *Ae. albopictus* and *Toxorhynchites amboinensis* (http://www.lgcstandards-atcc.org). The *An. stephensi* MSQ43 cell line is available from the Malaria Research and Reference Reagent Resource Center (www.mr4.org). Requests for mosquito cell lines can also be sent to the World Reference Centre for Emerging Viruses and Arboviruses (http://www.niaid.nih.gov/labsandresources/resources/dmid/wrceva).

### Application of mosquito cell lines

*In vitro* serial cell line passage experiments can be used to determine the host range of arboviruses in mosquitoes, ticks and vertebrates [39, 40]. In addition, insect cell lines can be used for protein expression analysis due to the post-translational processing and yield of expressed proteins in these eukaryotic cells. Various aspects of virus-mosquito host interactions can be investigated without the need for maintenance of insect colonies. For example, C6/36 mosquito cells have been used to demonstrate the cell entry mechanism of DENV [41]. Furthermore, defective DENV genomes have been detected in persistently infected C6/36 cells, providing valuable insight into the mechanisms through which arboviruses establish and maintain *in vivo* infections [42]. The antiviral immune response of *Ae. aegypti* to DENV infection has also been characterized in the immune-competent Aag2 cell line. Using transcriptomic analysis, it has been shown that DENV is capable of actively suppressing the mosquito immune response in infected cells [43]. Similarly, viral PIWI-interacting (pi)RNA-like molecules are produced following infection of mosquito cell lines with the mosquito-borne Semliki Forest virus, and have been shown to have anti-viral effects [44].

*In vitro* studies could be used to provide preliminary evidence for the potential of some mosquito species and vertebrate hosts to support replication of emerging arboviruses. For example, Rabensburg virus (RABV), a *Flavivirus* isolated from *Cx. pipiens,* was first considered to be a potential new lineage of WNV [45], but it is now postulated to be an intermediate between the mosquito-specific and horizontally transmitted flaviviruses [46]. RABV did not infect mammalian or avian cell cultures but efficiently infected mosquito cells [46]. Furthermore, although *Cx. pipiens* mosquitoes support replication of RABV, peroral transmission of infectious RABV was much lower, and vertical transmission higher, compared to WNV. Additionally, experimentally inoculated avian hosts did not become infected [46]. However, caution must be taken as *in vitro* vector-virus interaction may not always reflect *in vivo* vector competence or disease transmission in mosquito field populations.

Mosquito cell lines are also used extensively to passage arboviruses for *in vivo* transmission assays that involve collection of mosquito saliva during expectoration. Initially cell lines are used to amplify arbovirus titers required for oral feeding experiments. Mosquitoes presented with an arbovirus-infected blood meal undergo ‘forced salivation’, typically 7-14 days post-infection, to measure the length of the EIP of arboviruses within mosquitoes. Following forced salivation, mosquito cell lines such as C6/36 are inoculated with the collected saliva. Incubation of cells is then followed by an antibody-based detection protocol to determine whether infectious virus is present in saliva or through the appearance of plaque forming units. For low virus titers in saliva, several rounds of cell line passage may be undertaken to increase sensitivity.

Mosquito cell lines have also been used to determine the efficacy of novel compounds such as insecticides and biocontrol agents. Cell-based screening platforms have been developed to identify new compounds that are lethal to mosquito cell lines but show little or no activity against other insects (such as *Drosophila*) or human cell lines. Mosquitocidal cytotoxins were shown to be toxic to mosquito cells but not to *Drosophila* cells [47]. The gram positive bacterium *Bacillus thuringiensis israelensis* (Bti) produces insecticidal toxins active against mosquitoes that can be an effective larvicide for mosquito biocontrol. Cry4B is one of the major toxins produced by Bti and *Aedes* cell lines were used to demonstrate that Cry4B binds to several midgut membrane proteins including prohibitin, a protein recently identified as a receptor for entry of DENV into *Aedes* cells [48]. This study also utilized *Aedes* cells to show that pre-exposure to Cry4B results in a significant reduction in the number of infected cells compared to mock-exposed cells.

Mosquito-only flaviviruses (MOFs) have no known vertebrate reservoir host and have only been identified in mosquitoes. Cell lines have played an important role in the isolation and characterization of MOFs. The cell fusing agent virus was isolated in 1975 from an *Ae. aegypti* cell line [49]. Additional MOFs include Quang Binh virus isolated from *Cx. tritaeniorhynchus* [50], Kamiti river virus from *Ae. macintoshi* [51, 52] *,* Nakiwogo virus from *Mansonia africana* [53] and Calbertado virus from *Cx. tarsalis* [54–56]. Six potential MOFs were isolated from European mosquito species including five viruses that have not previously been reported in *Ae. caspius*, *Cx. theileri*, *Ae. vexans and Ae. cinereus* mosquitoes [57]. Inoculation of mosquito cell culture is often used to determine the presence of flavivirus isolates from wild mosquito material. A new virus, tentatively named Palm Creek virus, was isolated from *Coquillettidia xanthogaster* mosquitoes in Australia using infection of cultured mosquito cells [58]. Recently *Aedes* flavivirus (AEFV) strain SPFLD-MO-2011-MP6 was isolated in C6/36 cells from a pool of male *Ae. albopictus* mosquitoes that were reared to adults from larvae collected in the USA [59]. AEFV does not replicate in vertebrate cells, which is consistent with the lack of a vertebrate host range. Recently a new MOF from *Ochlerotatus caspius* mosquitoes was identified in Portugal and is referred to as *Ochlerotatus* flavivirus (OCFV) [60]. OCFV was also isolated in a C6/36 cell line where it replicated rapidly but failed to replicate in mammalian Vero cells.

Along with mosquitoes, other arthropods may also provide a useful source of cell lines for *in vitro* studies. *Culicoides* midges are important vectors of arboviruses such as bluetongue virus (BTV). Studies in *Culicoides* KC cell lines have demonstrated a functional RNAi response that inhibits BTV infection [61]. Tick cell lines have also been generated and have been used to study the response to tick-borne arboviruses of medical and veterinary importance. The susceptibility of cell lines from different tick species to Crimean-Congo hemorrhagic fever virus (CCHFV) revealed that there may be species-specific susceptibility to CCHFV infection [62].

### Cell lines from UK mosquito species

**Figure 2:**
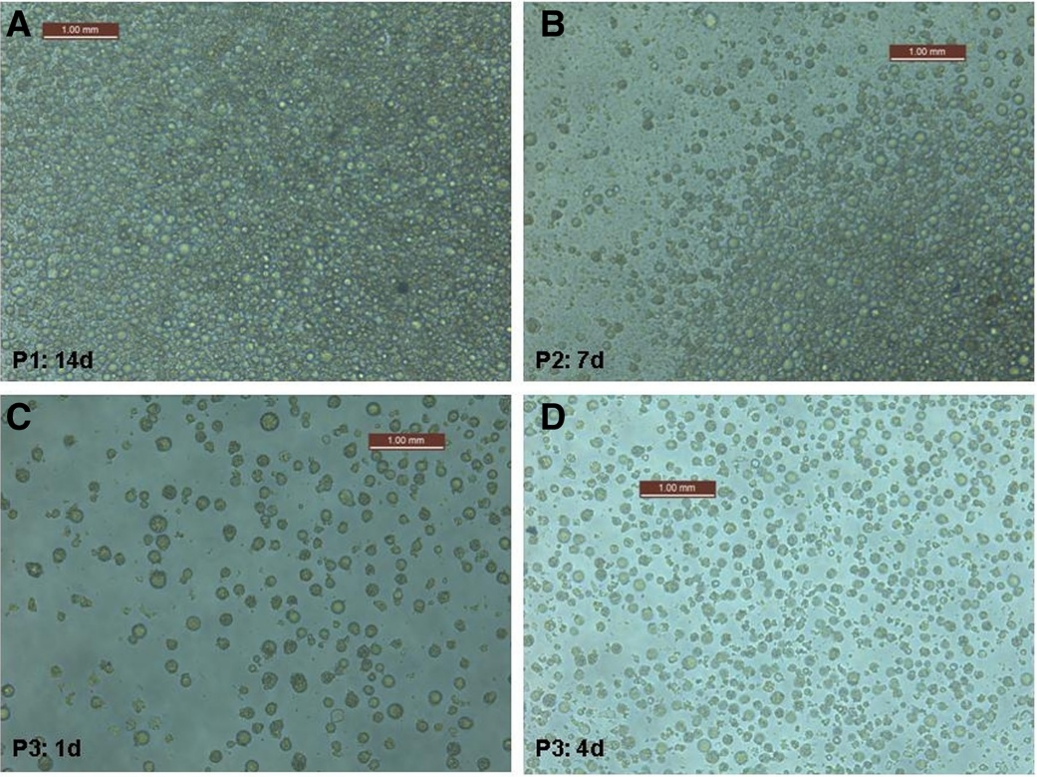
**Generation of an** ***Aedes vexans*** **cell line of UK origin using embryos as the source material.** The first passage (P) was undertaken 14 days (d) after adding crushed embryos to cell media and P2 was undertaken 7d after P1 to allow cells to adhere. Routine passage of the cell line was undertaken from P3 by transferring 20% cells in media to 80% media. **A)** P1 after 14d showing formation of a high density monolayer. **B)** P2 after 7d showing the start of a monolayer forming. **C)** P3 after 1d and **D)** P3 after 4d showing growth of the cell line.

Although several mosquito cell lines have been established from major mosquito disease vectors such as *An. gambiae* (malaria) and *Ae. aegypti* (dengue), further cell lines are needed for native temperate mosquito species that are considered potential vectors of arboviruses. Sporadic WNV outbreaks have occurred in warmer regions of Europe for the past 20 years and could occur more frequently as a result of climate change [63]. *Cx. pipiens* mosquitoes are widespread throughout Europe, and are critical vectors of WNV in the USA and southern Europe. Currently available cell lines, such as C6/36 and RML-12, are high passage cell lines, which may not be susceptible to many arboviruses that are considered a threat to countries such as the UK, including WNV. *Ae. vexans* is a vector of Tahyna virus and is the most common mosquito in Europe, but is relatively rare in the UK. This species has also been shown to be a competent vector of both WNV and RVFV under laboratory conditions. Preliminary experiments have been undertaken to generate an *Ae. vexans* cell line from a colony of UK origin. As shown in Figure 2, evidence for cell line growth and adhesion to form a monolayer has been observed in early passages. This cell line will be used to determine the initial virus-vector interactions of *Ae. vexans* to a diverse range of arboviruses that are considered to be a potential threat to the UK and help to inform future *in vivo* vector competence studies.

There are over 30 endemic mosquito species in the UK and several, including *Cx. pipiens*, which are potential WNV bridge vectors from birds to humans [64]. Furthermore, 12 species native to the UK, including *Ae. vexans* and *Cx. modestus*, have been shown to transmit WNV in other countries. Any change to the UK climate that causes an increase in mean temperature or prolonged periods of above-average warm weather may potentially increase the geographical distribution of European mosquito-borne diseases such as WNV. As a result, studies to determine the vector competence of mosquito species that are most likely to serve as bridge vectors for WNV are required to assess the likelihood of introduction and transmission in the UK. Investigation using mosquito cell lines will therefore provide a relatively simple method for deriving preliminary data on a range of virus-vector interactions with UK mosquito species. Cell lines that are shown to not be susceptible to certain viruses likely indicate that *in vivo* vector competence will be limited for that mosquito species. The genetic background of mosquito host vectors influences the vectorial capacity. Specific vector genotype x virus genotype (G x G) interactions may promote adaptation of viral lineages to local mosquito vector genotypes [65]. The virulence of arboviruses is particularly dependent on the mosquito host genetic background but can also influence host fitness. A trade-off hypothesis of virulence has been shown to significantly influence the evolution of arboviruses [66]. Virulence is also influenced by the genetic diversity of arboviruses. The introduction of genetic changes to arbovirus genomes that leads to genetic variation is thought to be limited by the requirement to replicate in both vertebrate and invertebrate hosts. Mosquito cell lines under laboratory conditions can be used to overcome this limitation allowing the introduction of genetic changes and determination of the effect on virulence. Alternative replication can be manipulated using both mammalian and mosquito cell lines. For example, passing Venezuelan equine encephalitis virus through cell lines resulted in nucleotide and amino acid changes but no significant change in virulence was observed [67]. Passing JEV under the same conditions resulted in 22 nucleotide and amino acid changes and attenuated virulence, with genetic changes occurring within 5 passages [67]. The evolution of vesicular stomatitis virus in either mammalian or insect cells was compared to alternating passages, with 7 mutations accumulating in alternated passages, compared to 2-4 for the constant cellular environment [68]. However, the authors of this study concluded that the slow rates of evolution observed in natural arbovirus populations are not necessarily due to the need for the virus to compromise between adaptation to both arthropod and vertebrate cell types. In contrast, a study carried out with DENV in a human cell line (Huh-7), the C6/36 mosquito cell line and alternating passages revealed that mutations accumulated more rapidly in viruses passed in Huh-7 cells than in those passed in C6/36 cells or in alternation [69]. Mosquito cell lines and alternating passages with mammalian cells can play a key role in trying to understand the patterns of arbovirus genetic evolution.

## Conclusions

Experiments to determine virus-host interactions, with an appropriate cell line of UK genetic background, are necessary to assess the likely impact of the introduction of arboviral transmission in a new area and the ability of the pathogens to become established in the local mosquito population. The presence of insect-specific flaviviruses, particularly in mosquito populations, may also influence transmission of arboviruses to humans. For example, a laboratory colony of *Cx. pipiens* established from Colorado, USA, has been shown to be infected with a *Culex* flavivirus (CxFV) and early suppression of WNV replication was observed [70]. However, another study demonstrated that CxFV had no significant impact on WNV replication, infection, dissemination or transmission in *Cx. quinquefasciatus* mosquitoes [71]. Establishment of cell lines from native UK *Cx. pipiens* and *Ae. vexans* mosquitoes would provide suitable models to investigate insect-specific flaviviruses that are present in UK populations. Infection of cell lines with arboviruses such as WNV would provide valuable information to inform vector competence studies of these native mosquito species.

## Abbreviations

RNAi: RNA interference
EIP: extrinsic incubation period
DENV: dengue virus
*An.*: *Anopheles*
*Ae.*: *Aedes*
*Cx.*: *Culex*
WNV: West Nile virus
JEV: Japanese encephalitis virus
EEEV: Eastern equine encephalitis virus
LACV: La Crosse virus
RVFV: Rift Valley fever virus
Bti: *Bacillus thuringiensis israelensis*
MOFs: Mosquito-only flaviviruses
AEFV: *Aedes* flavivirus
OCFV: *Ochlerotatus* flavivirus
BTV: Bluetongue virus
CCHFV: Crimean-Congo hemorrhagic fever virus
CxFV: *Culex* flavivirus.
